# The porcine translational research database: a manually curated, genomics and proteomics-based research resource

**DOI:** 10.1186/s12864-017-4009-7

**Published:** 2017-08-22

**Authors:** Harry D. Dawson, Celine Chen, Brady Gaynor, Jonathan Shao, Joseph F. Urban

**Affiliations:** 1 United States Department of Agriculture, Agricultural Research Service, Beltsville Human Nutrition Research Center, Diet, Genomics and Immunology Laboratory, Beltsville, MD USA; 2 United States Department of Agriculture, Agricultural Research Service, Beltsville Agricultural Research Center, Molecular Plant Pathology Lab, Beltsville, MD 20705 USA

**Keywords:** Porcine, Database, Comparative genomics

## Abstract

**Background:**

The use of swine in biomedical research has increased dramatically in the last decade. Diverse genomic- and proteomic databases have been developed to facilitate research using human and rodent models. Current porcine gene databases, however, lack the robust annotation to study pig models that are relevant to human studies and for comparative evaluation with rodent models. Furthermore, they contain a significant number of errors due to their primary reliance on machine-based annotation. To address these deficiencies, a comprehensive literature-based survey was conducted to identify certain selected genes that have demonstrated function in humans, mice or pigs.

**Results:**

The process identified 13,054 candidate human, bovine, mouse or rat genes/proteins used to select potential porcine homologs by searching multiple online sources of porcine gene information. The data in the Porcine Translational Research Database ((http://www.ars.usda.gov/Services/docs.htm?docid=6065) is supported by >5800 references, and contains 65 data fields for each entry, including >9700 full length (5′ and 3′) unambiguous pig sequences, >2400 real time PCR assays and reactivity information on >1700 antibodies. It also contains gene and/or protein expression data for >2200 genes and identifies and corrects 8187 errors (gene duplications artifacts, mis-assemblies, mis-annotations, and incorrect species assignments) for 5337 porcine genes.

**Conclusions:**

This database is the largest manually curated database for any single veterinary species and is unique among porcine gene databases in regard to linking gene expression to gene function, identifying related gene pathways, and connecting data with other porcine gene databases. This database provides the first comprehensive description of three major Super-families or functionally related groups of proteins (Cluster of Differentiation (CD) Marker genes, Solute Carrier Superfamily, ATP binding Cassette Superfamily), and a comparative description of porcine microRNAs.

**Electronic supplementary material:**

The online version of this article (doi:10.1186/s12864-017-4009-7) contains supplementary material, which is available to authorized users.

## Background

Swine are an important models for human anatomy, nutrition, metabolism and immunology [1–3]. Their organs are anatomically and histologically similar to humans as are their sensory innervation and blood supply [4]. Pigs are naturally susceptible to infection with organisms that are closely related or identical to those species infecting humans including helminths (*Ascaris, Taenia, Trichuris, Trichinella, Shistosoma, Strongyloides*), bacteria (*Campylobacter, Chlamydia, Eschericia coli, Helicobacter, Neisseria, Mycoplasma, Salmonella),* protozoans (*Toxoplasma*) and viri (*Coronavirus*, *Hepatitis E, Influenza, Nipah, Reovirus, Rotavirus)* [2, 5, 6]*.* The last 10 years has seen a boon in the development of genetically modified pig as models for human cardiovascular and lung disease, neurodegenerative and musculoskeletal disorders [7, 8] and cancer [9]. There is also a robust effort to develop pigs as sources for organs and tissues for human xenotransplantation [10].

Despite these potential strengths as a model, the lack of an annotated database for porcine gene and protein expression data is a limiting factor for translating findings in one species to another. Multiple online databases exist for the storage and retrieval of diverse bovine, rodent or human biomedical data [11–19]. Other databases exist for Zebrafish (ZFIN, [20]), *C. elegans* (WormBase, [21]), and *Drosophila melanogaster* (Flybase, [22]). Databases that encompass multispecies analysis such as Homologene and/or that rely on manual annotation such as InnateDb [23] include bovine but not porcine genes. Several porcine genome companion databases exist; however they lack robust manual annotation and are somewhat limited in scope or are infrequently updated [16–19]. Agbase, a large, multispecies functional analysis database allows the user to search 51,489 porcine genes based on 12 criteria including gene and protein names (UniProt) and Gene Ontology (GO) annotations. Furthermore, databases can contain a significant number of errors due to their primary reliance on machine-based annotation [24]. For example, the SUS-BAR database [19] is designed to identify protein orthologs based upon data that includes annotations from the machine-annotated NCBI genome. NCBI has recently begun to include GO annotations into curated entries for non-human and rodent species but most of these are indirect and often based on observations made in other species. As swine are an important model for comparative human studies, there is a critical need to have a centralized, manually-curated source of information for biomedical research. To address these needs, we created the Porcine Translational Research Database.

## Construction and Content

To generate content of immunological relevance, broad-based literature searches were conducted using the following terms: apoptosis, B cell development or activation, CD markers, chemokines, chemokine receptors, cytokines, cytokine receptors, dendritic cells, type 1 IFN induced genes, inflammation, nuclear factor kappa-light-chain-enhancer of activated B cells (NFκ-B) signaling pathway, toll receptor signaling pathway, T cell development or activation, Th1 cell development and Th2 cell development. In addition, immunologically related genes associated with the susceptibility to or pathology of allergy, asthma, arthritis, atherosclerosis and inflammation were included. In addition, The Gene Ontology consortium’s community annotation wikis for immunology, cardiovascular disease and muscle biology were searched (http://wiki.geneontology.org/index.php/Main_Page). The Jackson Laboratory database of knockout mouse phenotypes was searched for genes leading to defects in immune or metabolic phenotypes when over or under expressed. These genes include the vast majority of genes that are related to immunity and inflammation [2, 3, 25, 26]. For additional metabolically related genes, genes involved in the transport or metabolism of macronutrients, trace vitamins and minerals were searched. Other genes, associated with the susceptibility to or pathology of atherosclerosis, diabetes, and obesity, were identified. This process identified 13,054 candidate human, bovine, mouse or rat genes/proteins of interest used to select potential porcine orthologs by searching various online sources of porcine gene information. One to one orthology of protein coding genes were determined by protein structure similarity (best reciprocal BLAST hits) and the presence of a corresponding gene in the syntenic region of the human and or mouse genome. No 1:1 orthology could be established for members of some gene families including the Leukocyte Immunoglobulin-like Receptor (LILR) Killer Cell immunoglobulin-like Receptor (KIR), Carcinoembryonic antigen-related cell adhesion molecule (CEACAM) and Cytochrome P450 superfamilies. One to one porcine orthologs of human genes utilize the approved HGNC Name according to the International Society for Animal Genetics (ISAG) publishing guidelines. We defined pseudogenes by the criteria used by Ensembl and ENSCODE; namely the presence of one or more stop codons in the open reading frame that disrupt the protein structure, and (usually) a lack of intron structure at the genome level [27]. Pseudogenes are further classified into Processed, Duplicated, Unitary or Polymorphic categories [27].

### Sequence generation

**Fig. 1:**
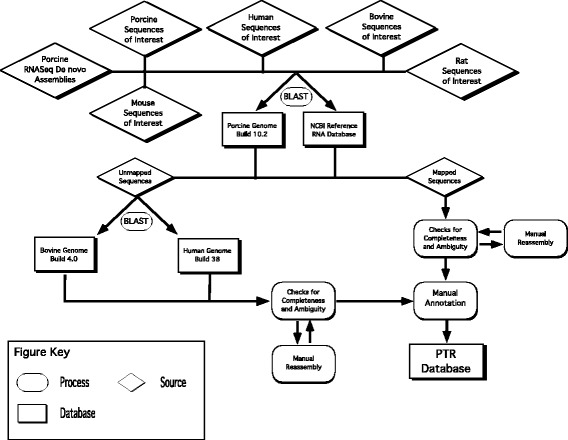
Porcine Translation Research Database (PTR) Construction Flowchart

Genbank (non-redundant, expressed sequences tag, high throughput genomic sequence, trace archive databases and whole genome shotgun contigs databases) was searched by discontiguous Megablast using default settings (word size = 11), using reference sequence accession numbers to human, bovine, mouse or rat genes/proteins of interest. A similar search was conducted in the following databases using the human or bovine reference sequence; NIH Intramural Sequencing Center (NISC) Comparative Vertebrate Sequencing Project [28]; National Center for Biotechnology Information (NCBI), *Sus scrofa* Genome Assembly releases 102 to 105 and Ensembl v10.2 releases 83 to 89. For genes that were determined to be missing from build 10.2 (Additional file 1) (and for the mis-assembled or duplicated gene artifacts (Additional file 1), we also constructed templates from de novo assemblies derived from Illumina 80 bp reads of the pig alveolar macrophage transcriptome (Dawson, unpublished results) using the de novo assembly algorithm of CLC Genomics Workbench using word size of 20 and a bubble size of 50. When necessary, predicted templates (from bovine or human sequences) were supplemented with porcine expressed sequence tag (EST) assemblies, single ESTs and portions of the published Tibetan (Bioproject # PRJNA291130), Wuzhishan (Bioproject # PRJNA144099), Goettingen (Bioproject # PRJNA291011) [29], Jinhua, Meishan, Bamei, Large White, Berkshire, Hampshire, Pietrain, Landrace, Rongshang and Duroc (Bioproject # PRJNA309108) porcine genomes [30]. ESTs were assembled using CAP3 (http://doua.prabi.fr/software/cap3). RNASeq reads were then mapped to these predicted templates in order to derive the full-length consensus sequence (unambiguous 6X coverage) using CLC Genome Workbench 7.0 (QIAGEN Bioinformatics, Redwood City CA). The following settings were used. Mismatch cost =2, Insertion cost = 3, Deletion cost = 3, similarity fraction = 0.95, length fraction = 0.95. Nucleotide sequences were translated using the ExPASy translate tool (http://web.expasy.org/translate/). A total of 1279 of these sequences have been deposited to the transcriptome shotgun assembly sequence database under Bioproject PRJNA80971 and the short read archive under project SRP013743). In silico-derived full-length RNA sequences are provided for an additional 3391 genes. This process/pipeline is summarized in Fig. 1. A summary of these sequences is provided in Table 1.

**Table 1:** Current Database Statistics (07/12/2017)

Parameter	Metric
Number of Entries	13,054
Number of Full-Length RNA Sequences (5′ and 3′ Representation)	9720
Number of Genes with Full-Length RNA Sequences	9165
Dawson Lab Full Length Submissions to Genbank	1351
Percent of Genome in Database with RNA Sequences	41.7
Number of Protein Coding Genes with Full-Length RNA Sequences	7805
Number of Protein Coding Gene Splice Variants	667
Number of Genes in Database with Full-Length Protein Sequences	8099
Percent of Genome in Database with Protein Sequences	42.6
Percent of Proteins in Database with Full-Length RNA Sequences	0.964
Number of Unigene Numbers Assigned	10,232
Unigene/Gene	1.45
Percentage of Entries with a Unigene Assignment	0.770
Entries with a Unigene Assignment	7056
Entries without a Unigene Assignment	2109
Number of NCBI Loci Represented	9967
NCBI Loci/Gene	1.088
Percentage of Entries with a NCBI loci Assignment	0.824
Entries with a NCBI loci Assignment	7549
Entries without a NCBI loci Assignment	1616

### Sequence analysis

**Fig. 2:**
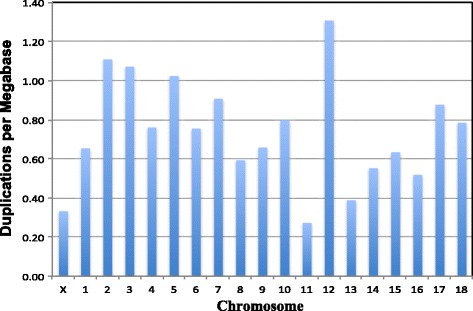
Chromosomal Locations of 1307 Duplicated Gene Artifacts (2889 Loci)

We randomly chose 268 of these mRNA for comparison of the 5′, 3′ and ORF length comparison to the corresponding human mRNA. Data are presented in Additional file 4. For the 1041 protein-coding genes missing from the genome, we entered the gene symbols into the DAVID version 6.8 (https://david.ncifcrf.gov) to assess overrepresentation of groups of gene with related function. The functional data were limited to human. Nine hundred and fifty six genes out of 1041 genes were recognized and 955 had functional annotations, of the unrecognized gene 41 are pig or artiodactyl specific genes. Data on functional enrichment of genes with a multiple comparison adjustment (Benjamini) value of >0.05 are presented in Table 3. We chose the 60 largest proteins of extreme size (>3000 amino acids) to compare the status (number of loci and completeness) in the NCBI and Ensembl build 10.2 genome. Because exon preservation is usually well conserved and there is fragmentation of certain areas of the porcine genome, the number of exons for the corresponding human gene was used for comparison. Lastly, we determined the chromosomal location of 1307 duplicated gene artifacts (2889 loci, Additional file 2) to identify problematic regions. Data are expressed as duplication per megabase (number of bases derived from the NCBI genome build (http://www.ncbi.nlm.nih.gov/genome?term=sus%20scrofa) and are presented in Fig. 2.

### Database implementation

**Fig. 3:**
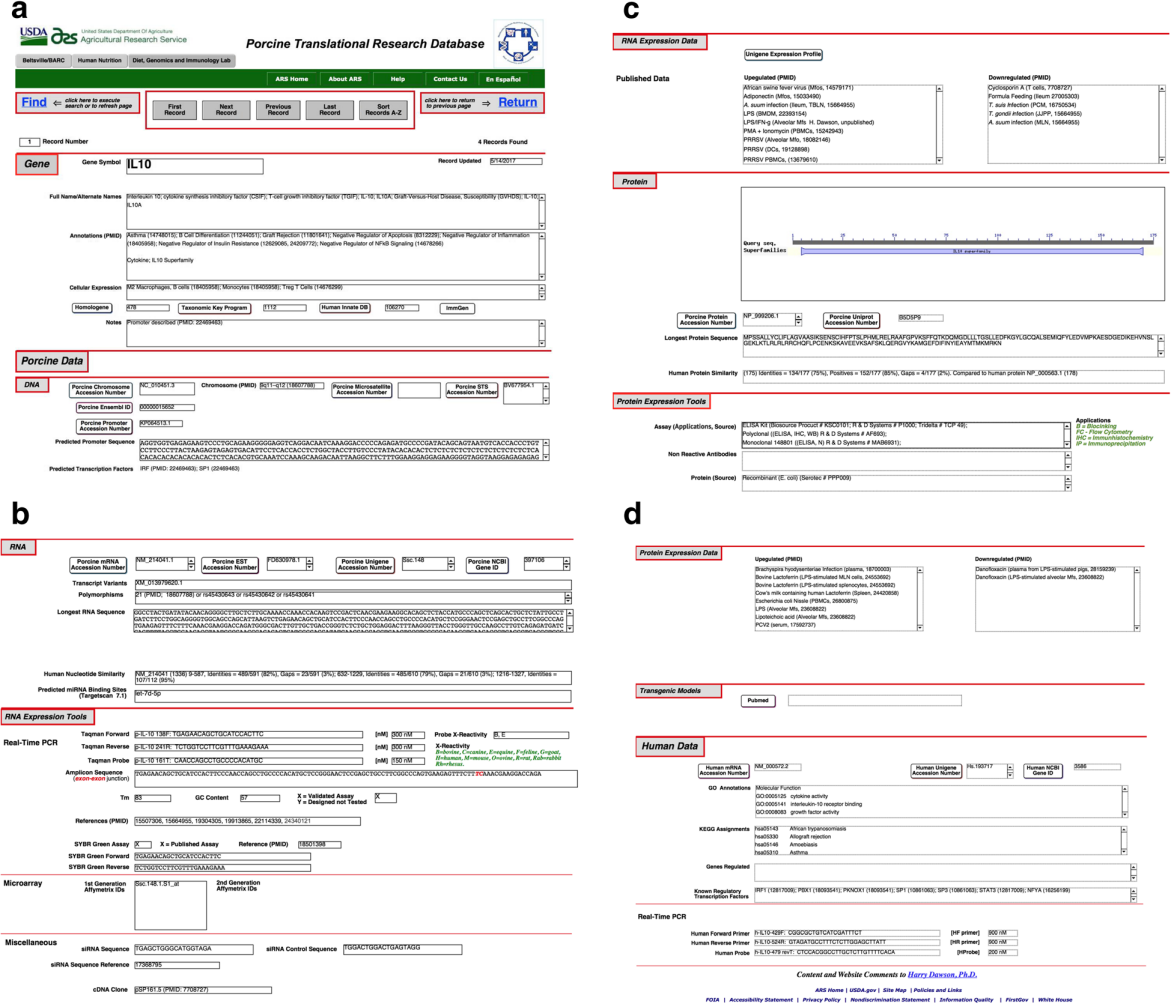
**a**–**d** Sample Database Entry

The currently described database was constructed in the Filemaker Po Advanced v14.0 program (Filemaker Inc., Santa Clara, CA). The layout is illustrated in the sample database entry for the cytokine IL10 (Fig. 3 panels A–D). It was deployed using the Filemaker Server Advanced v14.0 program (Filemaker Inc., Santa Clara, CA). External access to the database has been successfully tested using Chrome, Internet Explorer and Safari browsers. Other areas of the database were populated from existing published or our own unpublished data. Each publication is manually reviewed and data (antibodies, real-time PCR assays, RNA or protein expression data, functional data) is abstracted and entered into the database, along with the Pubmed ID, in the appropriate field. We have developed Taqman real-time PCR assays for 1867 of these genes making them cross reactive for as many species as possible (1067 are partially or fully human gene cross reactive). This is to ensure that comparable areas of the gene are being analyzed as well as for economic reasons. We also conducted a literature survey to determine the sequence of porcine SYBR green PCR assays. Tissue-specific gene expression summaries, using these assays, are provided for these and other studies (i.e., those using microarray and RNASeq), and a comprehensive search of catalog and published literature to identify antibodies to the corresponding proteins. Last, the “Notes Field” in the database was populated with information such as types of errors discovered, degree of 5′ and 3′ UTR conservation, degree of positive selection pressure in various species, and intron status. When the gene (sequence) is present in the genome but not annotated as a gene, we annotate the gene in the Notes field as “Not an identified gene in Ensembl build 10.2.” or “not an identified gene in NCBI build 10.2”.

**Fig. 4:**
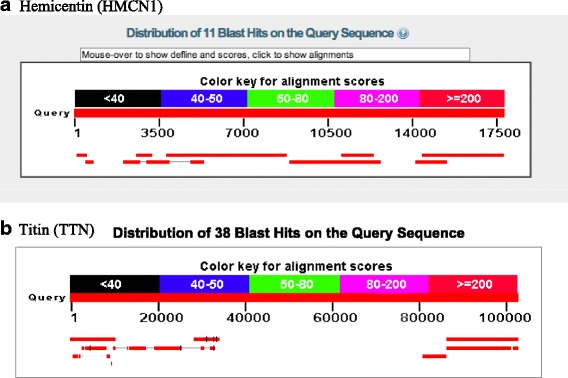
Hemicentin (﻿﻿**a﻿﻿**) and Titin (**b**) Assembly Blasts

**Fig. 5:**
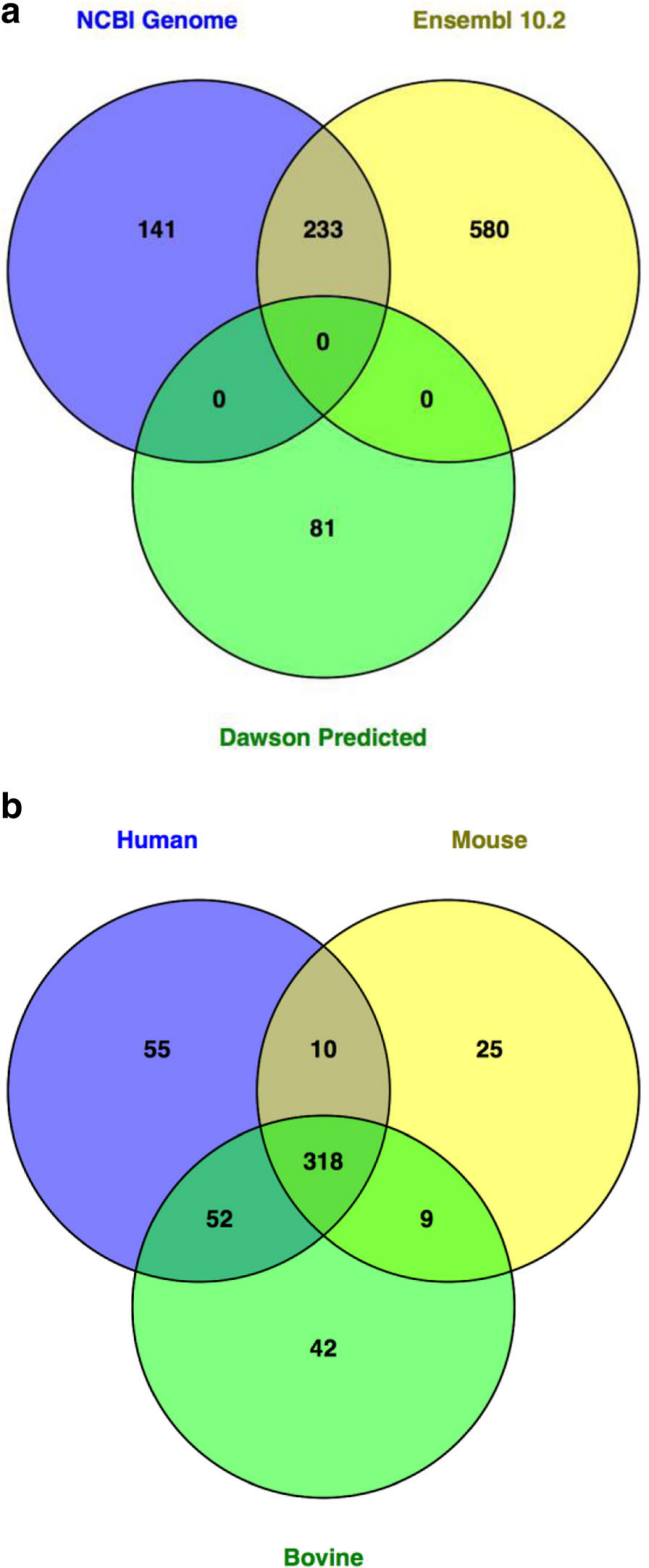
Analysis of MicroRNA Sequence Origin and Species Similarity. These 3 sources of information for our 1047 MicroRNA sequences have a significant amount of overlap (**a**) and include 81 that we have predicted based upon their presence in other species and other unfinished porcine genomes. Of these sequences, 454 are unique to pigs, 318 are shared among the four species (**b**), 55 are shared between humans and pigs but not mice and cows and 25 are shared between mice and pigs but not humans and cows

## Utility 

To date, we have generated 9720 full-length transcripts representing 9165 genes (Table 1). They include 1354 genes missing from Ensembl build 10.2 (Table 2 and Additional file 1) and 1400 genes that have been sequenced at least two times (gene duplicated artifacts shown in Table 2 and Additional file 2 that were annotated as separate genes in either Ensembl or NCBI builds. Functional enrichment analysis of 1041 protein-coding genes that are missing from the genome reveals that genes that are annotated as cytokines (24, *p* = 0.0053) and transcription factors (68) (particularly Homeodomain-like transcription factors (34, *p* = 0.032) and CENP-B/Helix-turn-helix (HTH) domains (6, *p* = 0.035) are significantly overrepresented (Table 3). Of note, the great majority of the Interleukin 1 Superfamily (IL1F10, IL1RN, IL36A, IL36B, IL36G, IL36RN, IL37) members are significantly (*p* = 0.0073) overrepresented. Data analysis that do not account for these genes risk missing assessment of important genes involved in inflammation and development.

**Table 2:** Number and Types of Errors Located in Publically-available Porcine Databases

Parameter	Metric
Number of Errors	8187
Number of Entries with Errors	5337
Number of Genes not identified in Ensembl Build 10.2.	1354
Missing from Genome	1019
Present but not Annotated	335
Artifactually Duplicated Loci	1400
Truncated proteins	2291
Elongated proteins	199

**Table 3:** Functional Annotations for 1041 Protein-Coding Genes that are Missing from Ensembl build 10.2

Category	Term	#	*P* Value	Benjamini
GOTERM MF DIRECT	Nucleic acid binding transcription factor activity	68	2.6E-3	3.0E-1
GOTERM MF DIRECT	Interleukin-1 receptor binding	7	1.8E-5	7.4E-3
GOTERM MF DIRECT	Cytokine activity	24	1.9E-5	5.2E-3
INTERPRO	Ly-6 antigen / uPA receptor -like	9	2.2E-6	2.9E-3
INTERPRO	Homeodomain-like	34	9.9E-5	3.2E-2
INTERPRO	DNA binding HTH domain, Psq-type	6	2.5E-4	6.2E-2
PFAM	CENP-B N-terminal DNA-binding domain	6	9.8E-5	3.5E-2
GOTERM BP	RNA biosynthetic process	241	4.5E-5	4.7E-2

Based upon gene number estimates from other closely related species such as human and cow, we estimated that our database has a coverage rate of approximately 42% of the porcine genome. These represent sequences found in 10,232 Unigene entries (1.45 per gene), 9967 NCBI loci (5756 are single loci that are not duplicated gene artifacts or split into multiple loci, and 1793 genes have multiple (4211) loci. A total of 2109 and 1616 of the genes have no assigned Unigene number or NCBI loci, respectively. In addition to GO and Kyoto Encyclopedia of Genes and Genomes (KEGG) annotations, literature-based functional annotations (derived from more than 5500 references) are provided for these sequences. We have also discovered a relatively large number (178) of porcine or artiodactyl-specific paralogs (Additional file 3) for 104 protein or non-protein coding porcine genes. For genes with multiple paralogs, genes are named in the order of phylogenetic distance of the parent human or bovine gene. Some of these genes are expressed pseudogenes. Some of these genes have been previously discussed (i.e., CD36, IL1B [25, 31]) or will be discussed in the following sections.

The transcripts we have generated for protein-coding genes include, on average, 70.5% of the corresponding 5′ and 3′ ends (each) of the human sequence (Additional file 4). The ORF is 99.4% conserved on a nucleotide count basis. These percentages indicate the fidelity of our procedure. We discovered extensive gene truncation (incomplete ORF) and gene duplicated artifacts (genes sequenced more than once) among the machine annotated versions of these genes. These problems are common among 1st drafts of other genomes [32, 33]. Gene duplicated artifacts appear most frequently for chromosomes 12, 2 and 3, and less frequently for chromosomes X, 11, 13, and 1 (Fig. 2). The most frequent areas should be targeted for re-sequencing or reassembly. Analysis of the 60 largest porcine proteins in the database shows that gene fragmentation and truncation roughly correlate with protein size and number of exons (Table 4). Figure 4 shows BLAST search results from two extremely large proteins, hemicentin (HMCN1, panel a) and titin (TTN, panel b) that have 9 loci assignments each, in the current NCBI build. Surprisingly, these proteins are not represented in Ensembl build 10.2 as annotated genes. Overall, of the 60 largest porcine proteins, only 6 and 10 are represented as single full-length sequences of the correct size in Ensembl and Genbank, respectively. We have deposited 12 de novo assemblies in the TSA archive and have provided in silico predicted RNA and protein sequences for 37 of these genes.

**Table 4:** Extensive Gene Fragmentation/Truncation Frequently Occurs Among Proteins of Extreme Size

Protein	Accession	# of Exons	Nucleotides	Amino Acids	NCBI Loci	Ensembl Loci
TTN	Predicted	312	103,020	33,921	9	0
SYNE1	Predicted	152	27,499	8798	3	3
OBSCN	Predicted	106	26,424	8755	5	2
MACF1	Predicted	141	23,519	7353	1 (truncated)	1 (truncated)
SYNE2	Predicted	116	21,767	6911	1	1
MUC6	Predicted	33	19,628	5692	1 (truncated)	1 (truncated)
MDN1	Predicted	102	17,684	5600	2	2
KMT2D	Predicted	56	17,324	5584	0	1 (truncated)
HMCN1	Predicted	107	17,839	5519	9	8
RNF213	Predicted	72	17,574	5245	4	3
UBR4	JAA53804.1	106	15,865	5182	4	4
RYR1	NP_001001534.1	106	15,384	5035	3	1 (truncated)
FAT4	Predicted	18	17,651	4983	4	1 (elongated)
RYR2	Predicted	107	16,588	4967	1 (truncated)	1 (truncated)
KMT2C	Predicted	64	15,669	4960	4	3
RYR3	Predicted	107	15,574	4870	8	1 (truncated)
BIRC6	Predicted	78	15,159	4861	2	1 (truncated)
HERC1	XP_001927286.4	80	15,199	4859	2	2
HERC2	JAG69485.1	98	15,070	4847	2	2
DNHD1	XP_013844910.1	41	16,014	4737	1	1 (truncated)
DNAH8	XP_001924974.2	97	14,418	4729	1	1
MYCBP2	Predicted	89	15,037	4675	1 (truncated)	1 (truncated)
DYNC1H1	Predicted	78	14,323	4646	3	1 (truncated)
LRP1B	Predicted	91	16,455	4590	13	3
FAT1	Predicted	29	14,904	4588	1	1 (elongated)
APOB	Predicted	31	14,158	4573	4	4
FAT3	Predicted	33	18,857	4557	1 (truncated)	1 (elongated)
LRP1	JAA53703.1	89	14,074	4544	2	2
ABCA13	Predicted	75	15,009	4444	3	0
SACS	Predicted	12	15,381	4441	4	3
ANK3	XP_005671069.1	52	15,032	4376	1	1 (truncated)
HUWE1	Predicted	91	14,590	4373	1 (elongated)	2
VPS13D	Predicted	70	16,126	4364	3	1 (truncated)
FAT2	Predicted	31	14,579	4350	2	1 (truncated)
PKD1	NP_001233131.1	47	14,212	4305	1	1
HECTD4	Predicted	76	19,834	4271	1 (truncated)	1 (truncated)
PRKDC	Predicted	86	14,362	4135	5	2
ANK2	Predicted	55	14,520	4100	1 (truncated)	1 (truncated)
VPS13B	JAG69054.1	80	13,584	3993	3	3
KMT2A	JAG69421.1	37	16,597	3967	1 (truncated)	1 (truncated)
DNAH12	Predicted	78	11,946	3961	3	1 (truncated)
AKAP9	NP_001240753.1	55	12,489	3898	1	1
LYST	JAA53665.1	61	12,677	3798	3	1 (truncated)
MUC4	XP_005670193.1	25	11,665	3745	1	1 (truncated)
ZNF469	Predicted	3	12,517	3736	0	0
VPS13C	JAA53695.1	88	11,772	3714	3	2
ZFHX3	Predicted	11	15,821	3713	1 (truncated)	1 (truncated)
DMD	NP_001012408.1	87	13,770	3674	5	2
SMG1	JAG69152.1	64	15,532	3659	2	0
SPEN	JAG69140.1	15	12,261	3655	1 (truncated)	1 (truncated)
CUBN	Predicted	71	11,536	3620	4	2
ZFHX4	Predicted	15	14,156	3611	1 (truncated)	1 (truncated)
WDFY3	XP_005656619.1	74	14,209	3594	1	1
USP34	JAA53700.1	80	11,327	3547	3	3
UTRN	JAA53694.1	84	10,547	3432	4	5
COL6A3	XP_013840079.1	50	13,801	3199	1	1 (truncated)
VPS13A	Predicted	76	11,078	3172	2	1
CEP350	JAA53656.1	40	9910	3121	2	2
CELSR1	Predicted	38	11,081	3031	2	1 (truncated)
FRY	Predicted	66	10,455	3016	3	2

In previous studies, we extensively compared porcine, human and mouse genes related to immunity and inflammation [2, 3, 25, 26]. In the following section, we will summarize our findings for three major Superfamilies or functionally related groups of proteins (CD marker genes, Solute carrier superfamily, ATP binding cassette superfamily) or non-coding RNA (microRNA) that have complete or nearly complete representation. CD markers (accessible as a group by entering CD markers in the Annotations field) encode a heterogeneous group of cell surface proteins. The Human Leucocyte Differentiation Antigen (HLDA) workshop has designated 408 molecules (some of which are grouped within a CD) as CD markers [34]. Based upon our assembly and analysis, we could establish 1:1 orthology for 357 porcine genes to those that compose HLDA version 10. Forty-three genes are not present in the porcine genome or could not be designated as 1:1 orthologs. Of these, nine genes (CLEC4C, CLEC4M, SIGLEC7, BTN3A1, LILRA1, LAIR2, PSG1, SIRPG, TNFRSF10C, are primate-specific [35–37]. KLRC2 (CD159c) is found in humans and rodents but not pigs. FCGR2C is a human-specific gene/pseudogene that belongs to a family of three low-affinity immunoglobulin gamma Fc receptors (CD32) [38]. We have determined that pigs have two member of this family that roughly corresponds to FCGR2A and FCGR2B. TNFRSF14 (CD270) is a marker for B cells, dendritic cells, monocytes, and Treg cells [39] found in humans and rodents, but not cows. Although, canine, feline, equine and ursine homologs have been identified, this gene may be a pseudogene in pigs as the putative ORF is interrupted by an endogenous retroviral sequence (H. Dawson, unpublished). FCRL2 (CD307b) is a marker for B cells in humans. Although sequences corresponding to FCRL2 have been identified in other mammals including dog and horse, no mouse ortholog has been identified [40]. This gene shows evidence of positive selection in humans [41] and is most likely a pseudogene in pigs.

Due to rapid evolution and post-speciation gene duplication, no 1:1 orthology could be established for most mouse and pig LILR or KIR family members, including LILRA4 (CD85G) and LILRB4 (CD85K) [42]. Similarly, other than CEACAM1 (CD66) and CEACAM6 (CD66C), no 1:1 orthology could be established for most pig and mouse CEACAM family members (CEACAM3 (CD66D) CEACAM5 (CD66E). CEACAM8 (CD67) may be a pseudogene as ESTs in Unigene Ssc.60435 predict a 243 amino acid protein interrupted by several stop codons. CEACAM8 and CEACAM6 were previously determined to have no direct murine orthologs [35]. Several other shared human-pig CD marker orthologs (ADGRE2 (CD312), ADGRE3 (CD313r), CD1A, CD1E, CR1 (CD35), CD58, FCGR2A (CD32), FCAR (CD89), FCRL3 (CD307c), FCRL4 (CD307d), ICAM3 (CD50), NCR2 (CD336), NCR3 (CD337) and TLR10 (CD290r) have no rodent orthologs [2, 40, 43].

A significant number of errors were discovered in genes encoding porcine orthologs of human CD markers; 25 are not present in Ensembl build 10.2, 88 of the proteins are truncated and 52 are duplicated gene artifacts. Sixty-seven full-length mRNA sequences encoding proteins, assembled from macrophage RNA-Seq reads, have been deposited in Genbank. An additional 79 in silico constructs are provided. Antibody data, gathered from publications, manufacturers or generated in house, is provided for 186 proteins including 395 monoclonal and 285 polyclonal antibodies. Additional cross reactivity for 29 proteins is expected because they are >95% similar to human proteins. Several of the CD Marker family are members of other gene families including the Solute Carrier and ATP-binding Cassette Super Family.

The Human Genome Organization’s gene nomenclature committee (HGNC) has assigned 395 genes to the Solute Carrier Superfamily, 21 are pseudogenes and three hundred seventy four encode proteins (accessible as a group by entering Solute Carrier Superfamily in the Annotations field). These are organized into 52 subfamilies; about 25% are dedicated to nutrient transport. The porcine Solute Carrier Super family contains 398 protein-coding members and all human subfamilies are represented. Forty-two of these genes are present in other porcine genomes but missing from Ensembl build 10.2, 113 are truncated and 58 of these are duplicated gene artifacts. Sixty three full-length mRNA sequences, assembled from macrophage RNA-Seq reads, have been deposited in Genbank and an additional 159 in silico constructs are provided. Forty-two of these genes are missing from all porcine genomes or are present as pseudogenes. Among these genes are UCP1 (thermogenein), a protein involved in non-shivering thermogenesis and a pseudogene in pigs [44] and SLC52A2, a primate specific riboflavin transporter [45]. Other species-specific genes include eight primate-specific (SLC2A14, SLC22A24, SLC35E2, SLC35G3, SLC35G4, SLC35G5, SLCO1B1, SLCO1B7), one human specific (SLC22A25) gene and 14 mouse or rodent-specific genes (Slc6a20b, Slc7a12, Slc21a4, Slc22a19, Slc22a21, Slc22a22, Slc22a26, Slc22a27, Slc22a28, Slc22a29, Slc22a30, Slco1a1, Slco1b2, and Slco6b1). SLC25A18 is present in human and rodent genomes but is missing from bovine and porcine genomes. SLC25A52 is present in primate and rat genomes but not mouse. SLC9C2 is a pseudogene in mouse [46]. SLC22A31 is an expressed pseudogene in pigs and is missing in rodents. SLC22A11 is an expressed pseudogene in pigs and a non-expressed pseudogene in mouse. Lastly, SLC23A4, an intestinal nucleobase transporter [47], is a pseudogene in humans but is present in pig, cow and rodent genomes. Several porcine or artiodactyl-specific gene expansions are found in subfamilies (Additional file 3) including SLC7A3 (14 members), SLC7A13 (3 members) SLC22A6 (2 members), SLC22A10 (4 members) and SLC47A1 (2 members). The biological functions of these paralogs remain to be determined; however the parent genes are involved in amino acid (SLC7A3, SLC7A13) or dipeptide transport (SLC22A6) [48, 49].

The HGNC has assigned 51 genes to the ATP binding Cassette Superfamily, three are pseudogenes and 48 encode proteins (accessible as a group by entering ATP binding Cassette Superfamily in the Annotations field). These are organized into five subfamilies (A-G), about 20% are dedicated to nutrient (i.e., carotenoid, cholesterol and vitamin A) transport. The porcine ATP binding Cassette Family contains 57 members and all human subfamilies are represented. These include five that are missing from Ensembl build 10.2 and 18 that are duplicated gene artifacts. Five of these genes are present in other porcine genomes, but missing from Ensembl build 10.2, 21 are truncated, and 18 of these genes are duplicated gene artifacts, Eleven full-length mRNA sequences, assembled from macrophage RNA-Seq reads, have been deposited in Genbank and an additional 24 in silico constructs are provided.

An analysis of this superfamily revealed that ABCC11 has no murine ortholog [50] and ABCA8 has no direct rodent ortholog as the gene has diverged into two paralogs, Abca8a and Abca8b [51]. The ABCC4, a prostaglandin E2 transporter [52], has diverged from the parent gene into five paralogs (ABCC4L1, ABCC4L2, ABCC4L3, ABCC4L4 and ABCC4L5 (Additional file 3). ABCA10, involved in human macrophage cholesterol transport [53], is a pseudogene in rodents. It may be an expressed pseudogene in pigs as the predicted protein is half (787 amino acids) the size of human ABCA10 (1543 amino acids) and weak expression (by RNASeq) was detected in macrophages and moderated expression in intestine (H. Dawson, unpublished). ABCA17 is an expressed pseudogene is humans and pigs. Like the Solute Carrier Superfamily, most of the genes in the ATP binding Cassette Super family have not been characterized at the functional level. Nevertheless, the similarities and differences in the ATP binding Cassette and ATP binding Cassette Super families impact the suitability of rodents and pigs as models for human drug and nutrient transport and metabolism.

The exact number of microRNAs in the porcine genome is unknown. There are 4272 annotated microRNAs in the human genome (build 30). Although there are several papers describing the measurement of porcine microRNAs in various tissues or estimating the number in the porcine genome [54–57] and three partially overlapping sources of porcine microRNA sequences, the exact number of porcine microRNAs is currently unknown. There are only 382, 385 and 816 (non-redundant) annotated pig miRNA sequences in Mirbase, NCBI gene build, and Ensembl build 10.2, respectively. These three sources of information have a significant amount of overlap (Fig. 5a). We have consolidated this information and provide sequence data for our own predicted sequences based on conserved sequence identity to 1900 human, mouse or bovine sequences, to provide 1033 non-redundant porcine microRNA sequences (accessible as a group by entering MicroRNA in the Annotations field). Of note, all of the sequences found in Mirbase were found in the NCBI gene build, 59 of the microRNA sequences in Ensembl were found to be duplicated artifacts, and 214 of the 1033 sequences are not present in the current Ensembl gene build (10.2). This includes 81 that we have predicted based upon their presence in other species and other unfinished porcine genomes. We discovered the following species- or genera-specific microRNA; pigs (454), humans (199), primates (111) bovine (179), mouse (76) and rodents (20). Many of the porcine-specific microRNA have arisen from biological duplication/expansion (Additional file 3). A comparison of microRNA that are present in pigs and shared among at least one of the three other species (human, cows, and mice) revealed that 318 microRNA are shared among the four species, 107 are shared between pigs, humans and cows but not mice, and 34 are shared between pigs, mice and cows but not humans (Fig. 5b). Thus, the frequency of non-conserved microRNA preservation between human and pig is nearly three times that of mouse to pig.

## Discussion

The Porcine Translational Research Database is named because of its unique utility to translate findings made in rodents to pigs and from those in pigs to humans. A comprehensive literature-based survey was conducted to identify genes that have demonstrated function in humans, mice or pigs. The resulting data in the database is documented by >6000 references. The database currently contains 65 data fields for each entry. Our efforts to improve the genome and its annotation are similar to other efforts, for example the sequencing of 12,000 genes to supplement annotation of the pig genome [32, 33, 58] and de novo assembly of multiple pig genomes to reveal 1737 protein coding genes that are missing from Ensembl build 10.2 [30]. The online Supplemental data from the latter manuscript was unavailable at the time of the preparation of this manuscript so no comparison could be made. The manual assembly of >9700 RNA sequences has direct practical implications for genomics-based analysis. The state of the current genome build (mis-annotations, duplication artifacts, and missing sequences) effectively prohibits its use for aligning RNAseq reads. We have used these sequences to compare gene expression separately from Ensembl 10.2 and have also compared the number of reads obtained from the corresponding templates in Ensembl 10.2. For the great majority of transcripts compared, as expected, our full-length sequences provided a higher level of sensitivity than the corresponding Ensembl sequences (H. Dawson unpublished).

The full 5′ and 3′ representation of each gene will also allow for characterization of regulatory regions and miRNA target sites. In our estimation, >40% of transcripts in Ensembl or NCBI genomes do not represent the full-length gene. Our efforts will also allow for further consolidation of porcine Unigene numbers. Currently, each gene is represented by from 0 to >10 Unigene assignments, and >10% of genes have more than one.

It is significant that we discovered a large number of errors (about 30% of entries) in the publicly available sequence databases (these can be accessed by searching the “Notes Field” using the word “error” (Fig. 3)). In addition to the duplication artifacts, mis-annotations and missing genes, we also encountered a number of RNA sequences in publically available archives belonging to other species. For, example, human (AHR, AF233432.1), panda (IL2, NM_001199892.1) and rat (NUDT14, ESTs in Unigene Ssc.85635) RNA sequences are annotated as porcine derived. We also found sources of contaminating DNA from completely unrelated species. For example, about 1/5 of porcine chromosome 4 clone CU076066.6 is from Zebrafish. These sequences represent 6 Zebrafish genes (LOC100003615, LOC447815, LOC108179932, LOC108183883, LOC108183971, and LOC103910681) and are annotated as porcine genes by Ensembl build 10.2 (ENSSSCG00000006223) and NCBI genomes (LOC100739857). Similarly, several NCBI loci (ASNA1L*, LOC100737282, LOC100737202, LOC100620149, LOC100737282) and one Ensembl locus (ENSSSCG00000026988) are derived from contaminating *Babesia bigemina* genomic DNA.

We have discovered several sources of systematic errors in the Ensmbl and NCBI gene/protein prediction or annotation pipelines. For example all selenoproteins in Ensembl are truncated because the codon (UGA) for selenocysteine is mistranslated or translated as a stop codon. We and others have identified a systematic error in the identification of another gene family, the Taste receptor, type 2 (TAS2R) Superfamily. Despite being intronless and mostly devoid of 5′ and 3′ UTR regions, Ensembl consistently fails to recognize them as genes [3]. These data illustrate the critical importance of the manual-curation process to reduce errors.

We believe that this is the largest manually curated database for any veterinary species and that the infomantics are unique among those targeting a veterinary species in regard to linking gene expression to gene function, identification of related gene pathways, and connectivity with other porcine gene databases, as well as for reagents that measure gene and protein expression. In addition, it is the largest source of centralized antibody information for the pig. Any database must be updated frequently in order to be useful. Currently the database is updated monthly and we anticipate expanding the content to include all porcine genes. There are several Super families of genes that will be the next targets of our efforts. One is the GPCR super family, the exact size of the GPCR super family is still unknown, but nearly 800 different human genes (or ~4% of the entire protein-coding genome) have been predicted to code for them. We will also continue to develop and annotate new assays. We intend to include our own prediction analysis for the promoter and 3′ UTR region of RNA for transcription factor and microRNA binding sites. Lastly, we intend to synchronize our database with the porcine “Snowball” array and porcine gene expression atlas [59].

## Additional files

Additional file 1: Porcine genes missing in Ensembl build 10.2 of the porcine genome. Gene names and evidence/source for RNA sequence of genes that are missing from Ensembl build 10.2. (XLSX 112 kb)

Additional file 2: Artifactually duplicated genes in Ensembl build 10.2. Gene names, Ensembl and NCBI loci numbers and NCBI genome build 10.2 coordinates of artifactually duplicated genes (XLSX 282 kb)

Additional file 3: Porcine or artiodactyl-specific paralogs. Gene names, Ensembl and NCBI loci numbers and Build 10.2 NCBI gene coordinates of porcine or artiodactyl-specific paralogs (XLSX 58 kb)

Additional file 4: 5′, ORF and 3′ end comparison of porcine and human mRNAs. 5′, ORF and 3′ end comparison of porcine and human mRNAs (XLSX 66 kb)
